# Effectiveness and Medicoeconomic Evaluation of Home Monitoring of Patients With Mild COVID-19: Covidom Cohort Study

**DOI:** 10.2196/43980

**Published:** 2023-06-23

**Authors:** Luc Jaulmes, Youri Yordanov, Alexandre Descamps, Isabelle Durand-Zaleski, Aurélien Dinh, Patrick Jourdain, Agnès Dechartres

**Affiliations:** 1 Centre de pharmaco-épidémiologie de l’APHP, Dépt. de Santé Publique, Hôpital Pitié Salpêtrière, Sorbonne Université, AP-HP Paris France; 2 Sorbonne Université, AP-HP, Hôpital Saint Antoine, Service d'Accueil des Urgences, INSERM, Institut Pierre Louis d'Epidémiologie et de Santé Publique, UMR-S 1136 Paris France; 3 CIC Cochin Pasteur INSERM CIC 1417, Université Paris Cité AP-HP Paris France; 4 Institut Pierre Louis d’Epidémiologie et de Santé Publique INSERM, Université Paris Est AP-HP Paris France; 5 URC Eco, Hôpital de l’Hôtel Dieu DRCI AP-HP Paris France; 6 Infectious Disease department, University Hospital R. Poincaré UVSQ AP-HP Garches France; 7 INSERM U999, CHU Bicêtre AP-HP Université Paris-Saclay AP-HP Gif-sur-Yvette France; 8 Sorbonne Université, INSERM, Institut Pierre Louis d’Epidémiologie et de Santé Publique, AP-HP. Sorbonne Université, Hôpital Pitié Salpêtrière, Département de Santé Publique, centre de pharmaco-épidémiologie de l’APHP, F75013 Paris France

**Keywords:** COVID-19, Covidom, home monitoring, telehealth, tele-surveillance, primary outcome, remote monitoring, digital health intervention, emergency medical service, patient care, digital care, mobile phone

## Abstract

**Background:**

Covidom was a telemonitoring solution for home monitoring of patients with mild to moderate COVID-19, deployed in March 2020 in the Greater Paris area in France to alleviate the burden on the health care system. The Covidom solution included a free mobile application with daily monitoring questionnaires and a regional control center to quickly handle patient alerts, including dispatching emergency medical services when necessary.

**Objective:**

This study aimed to provide an overall evaluation of the Covidom solution 18 months after its inception in terms of effectiveness, safety, and cost.

**Methods:**

Our primary outcome was to measure effectiveness using the number of handled alerts, response escalation, and patient-reported medical contacts outside of Covidom. Then, we analyzed the safety of Covidom by assessing its ability to detect clinical worsening, defined as hospitalization or death, and the number of patients with clinical worsening without any preceding alert. We evaluated the cost of Covidom and compared the cost of hospitalization for Covidom and non-Covidom patients with mild COVID-19 cases seen in the emergency departments of the largest network of hospitals in the Greater Paris area (Assistance Publique-Hôpitaux de Paris). Finally, we reported on user satisfaction.

**Results:**

Of the 60,073 patients monitored by Covidom, the regional control center handled 285,496 alerts and dispatched emergency medical services 518 times. Of the 13,204 respondents who responded to either of the follow-up questionnaires, 65.8% (n=8690) reported having sought medical care outside the Covidom solution during their monitoring period. Of the 947 patients who experienced clinical worsening while adhering to daily monitoring, only 35 (3.7%) did not previously trigger alerts (35 were hospitalized, including 1 who died). The average cost of Covidom was €54 (US $1=€0.8614) per patient, and the cost of hospitalization for COVID-19 worsening was significantly lower in Covidom than in non-Covidom patients with mild COVID-19 cases seen in the emergency departments of Assistance Publique-Hôpitaux de Paris. The patients who responded to the satisfaction questionnaire had a median rating of 9 (out of 10) for the likelihood of recommending Covidom.

**Conclusions:**

Covidom may have contributed to alleviating the pressure on the health care system in the initial months of the pandemic, although its impact was lower than anticipated, with a substantial number of patients having consulted outside of Covidom. Covidom seems to be safe for home monitoring of patients with mild to moderate COVID-19.

## Introduction

### Background

As of July 2022, the pandemic caused by SARS-CoV-2 resulted in an estimated 541 million cases and 6.3 million deaths worldwide [1]. With severe forms of COVID-19 requiring hospitalization and sometimes intensive care unit admission with mechanical ventilation, hospitalization and critical care saturation were major concerns [2]. To mitigate these risks, governments worldwide, including France, implemented solutions ranging from prohibiting large gatherings and mandating surgical masks to complete lockdowns [3,4]. Policies also included setting up digital tools, ranging from contact tracing to monitoring of quarantined patients [5,6].

Most COVID-19 infections are mild to moderate or even asymptomatic [7,8]. The Covidom telemonitoring solution was rapidly deployed in March 2020 in the Paris region for home monitoring of patients with mild to moderate COVID-19, with the aim of alleviating the burden on hospitals and general practitioners and reducing the risk of transmission thanks to safe home isolation [9]. This solution consisted of a free mobile application sending daily monitoring questionnaires and a regional control center with remote monitoring responders (RMRs) and physicians to handle the alerts generated by an algorithm based on patients’ answers to the questionnaires. Whenever an alert was generated, the regional control center systematically called back the patient for a remote assessment and proposed a medical response if necessary, ranging from a teleconsultation to sending emergency medical services (EMS) at home.

### Objectives

In this study, we aimed to provide an overall evaluation of the effectiveness of Covidom 18 months after its inception in terms of the number and type of alerts handled and safety, including the ability of alerts to detect patients with clinical worsening. We also reported an assessment of Covidom from an economic perspective and patient satisfaction.

## Methods

### Overview of the Covidom Solution

The Covidom solution was presented in previous papers [9,10]. In short, Covidom was a telemonitoring solution for patients with a suspected or confirmed mild to moderate case of COVID-19. Covidom included a mobile application sending daily questionnaires and a regional control center dealing with the alerts generated by the patients’ answers to the questionnaires.

Patients were included in Covidom by a physician, in most cases as outpatients at the end of a medical encounter for symptoms evocative of COVID-19 or at discharge after a COVID-19–related hospitalization. Patients first received brief information from the physician and provided oral consent to being included, after which they received a link to complete registration. Once registered, patients answered an *inclusion questionnaire* on comorbidities and symptoms and then daily *monitoring questionnaires* up to 30 days after the onset of the first symptoms or after hospital discharge. For patients considered by the physician as high risk (in case of cardiovascular disease, diabetes, chronic lung disease, immunodeficiency [transplant, active cancer treatment, uncontrolled HIV infection, etc], third trimester of pregnancy, or age >65 years), the number of daily monitoring questionnaires was 2 instead of 1.

On days 14 and 30 after symptom onset, patients received *follow-up questionnaires* that provided further information on symptom evolution, diagnostic confirmation, the use of care, and patient satisfaction. The questionnaires have been included for reference in Multimedia Appendix 1.

### Ethics Approval

All patients were informed at registration of the potential use of their anonymized data for research purposes. This study was conducted on the anonymized data of patients who did not oppose research use and was approved by the Scientific and Ethical Committee of Assistance Publique-Hôpitaux de Paris (AP-HP; IRB00011591). Patients received no compensation, monetary or otherwise, for inclusion in the study.

### Patients Included in This Study

We included all patients aged ≥18 years with suspected or proven COVID-19 as evaluated by the recruiting physician; who completed Covidom registration; and who had a date of COVID-19 symptom onset earlier than November 1, 2021. We also considered suspected cases, as during the first months of the pandemic, positive reverse transcription polymerase chain reaction (RT-PCR) tests were not mandatory in the French health authorities’ case definition. At that time, RT-PCR testing was not widely available and was reserved for the most severe cases.

Eligible patients were matched with 2 complementary databases: the AP-HP data warehouse (Entrepôt de données de santé de l’AP-HP) and the French National Institute of Statistics and Economic Studies lists of deaths [11]. AP-HP is a network of 39 university hospitals in the Greater Paris area, covering a large part of the population in this area (12 million inhabitants). This matching allowed us to complement the information on diagnostic confirmation, possible hospitalizations, and death during follow-up.

In this study, we reported the general characteristics (age and sex), as recorded by the physician for all included patients, and the detailed characteristics, including comorbidities and symptoms, for patients who completed the inclusion questionnaire.

### Alerts Generated and Response

The daily monitoring questionnaires could trigger alerts, which were either *normal priority* or *high priority*, based on the number and intensity of symptoms reported by the patient (detailed in Multimedia Appendix 1). The regional control center systematically called back patients to handle each alert appropriately, which may include simple reassurance, remote medical assessment, addressing the patient to their general practitioner (GP), to a hospital emergency department (ED), or dispatching EMS to the patient’s home. For each alert, we recorded the time to first response and the duration until the resolution of the alert, which we called handling time.

The RMRs took notes on each alert handled and classified the alerts using labels. The main types of labels are presented in Textbox 1.

Labels were progressively added over time as patterns emerged in the handled alerts. To account for this, we reported the number of times each label appeared and computed the label frequency starting 30 days after its first use. For the highest level of escalation only (control center physicians called EMS), we reported the number and frequency over the entire study period. This was limited to this single label, as it required manual screening of all alert reports before label introduction. We also reported the distribution of false alerts to understand the possible causes.

**Main types of labels.**

**Levels of escalation:**

- Patient addressed to a general practitioner
- Call forwarded to control center physicians
- Patient addressed to an emergency department (ED)
- Control center physicians called emergency medical services (EMS)

**Consultations at the patient’s initiative, reported in the conversation:**

- Spontaneous ED consultation
- Spontaneous call to EMS
- Hospitalization

**Other information:**

- The patient requested psychological support
- False alert: incorrect measures (of respiratory rate, temperature, etc).

### Primary Outcome

Our primary outcome was the effectiveness of Covidom, that is, the extent to which the solution fulfilled its objectives in practice [12]. To quantify effectiveness, we reported the number and type of alerts handled by the regional control center. Label frequencies were used to estimate the total number of alert types over the study period.

### Evaluation of Covidom Safety

To assess the safety of Covidom, we considered alert handling time by type of alert, patient clinical worsening, and medical contacts outside of Covidom, reported by the patients in their follow-up questionnaires. We evaluated the time to alert first response and the time to alert resolution by comparing the values for both normal and high-priority alerts. In addition, we compared the rate of EMS dispatch events in high- and normal-priority alerts to assess the pertinence of priority classification.

Clinical worsening was defined as either hospitalization (or, in the case of patients included at hospital discharge, rehospitalization) or death within 30 days after the date of first symptoms and hospital discharge, respectively. This information was obtained either from hospitalization data by matching with the AP-HP data warehouse, from responses to follow-up questionnaires, or from end-of-monitoring reasons when RMRs systematically called back patients who stopped responding to their daily monitoring questionnaires. We also reported separately the rates of hospitalization (for outpatients), rehospitalization (for patients included at hospital discharge), and death. We screened the alerts generated by patients who presented with clinical worsening and whose clinical worsening date was known and reported the proportion of those patients who had no alerts in the 5 days preceding the worsening, as well as their adherence to the Covidom solution.

### Economic Assessment of Covidom

We reported the total cost of operating Covidom, including costs related to regional control center, RMRs, and technical support (eg, application development), divided into staff, information technology, and structural and administration costs. We then estimated the health care costs of patients included in Covidom by computing both out-of-hospital and hospitalization costs. The out-of-hospital costs were obtained from the medical contacts reported in day-14 and day-30 follow-up questionnaires and from medical contacts initiated by RMRs. These included consultations, calls to EMS regulation centers, ED visits, and EMS dispatches. The unit costs and sources for cost computations are presented in Multimedia Appendix 2 [13-15]. Hospitalization costs were estimated for patients included in Covidom and those hospitalized in one of the AP-HP hospitals, as identified through data matching with the AP-HP data warehouse. The diagnosis-related group classification of each hospitalization was obtained from the AP-HP data warehouse. We estimated the cost of each hospitalization from the national average cost per diagnosis classification according to the latest schedule of reference costs [16] and from the observed length of hospital stay.

### Comparison Among Mild COVID-19 Cases Presenting at AP-HP EDs

Although it was impossible to compare the total health care costs of patients with mild COVID-19 included in Covidom with a similar population not included in Covidom, the AP-HP data warehouse allowed us to compare the delay until hospitalization and hospitalization costs between these populations. We considered all patients who had an initial consultation for COVID-19 at an AP-HP hospital ED and who were not hospitalized within 24 hours of this initial consultation as mild COVID-19 patients at the time of the consultation and thus eligible to be included in Covidom. We reported the number, age, and sex of these patients. For patients who were later hospitalized, we compared the delay in days from ED consultation to hospitalization and the hospitalization costs between patients effectively included in Covidom and those who were not.

### Patient Satisfaction

Finally, we reported the answers to the satisfaction questions in the follow-up questionnaires (see the full text in Multimedia Appendix 3). These questions were asked in the 30-day follow-up questionnaire from April 10, 2020, to May 13, 2020, and in the 14-day follow-up questionnaire since May 14, 2020, to enhance the response rate. Patients were asked to rate several questions on a scale from 0 to 10, with higher scores indicating greater agreement with the question. The questions were related to the understandability of the monitoring questionnaire, interface usability, patient experience with Covidom as a type of care, whether they would recommend Covidom, whether they felt supported psychologically, and whether Covidom helped reduce stress. We described the median score with IQR for each question.

## Results

### Patient Characteristics

**Figure 1:**
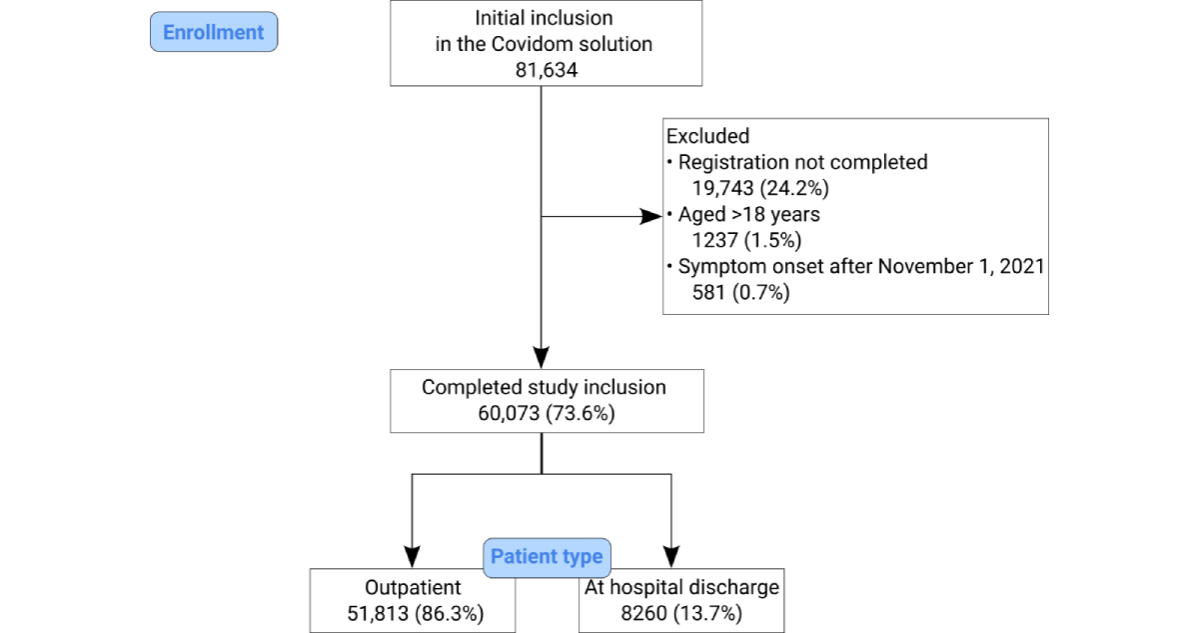
Flowchart of patients registered on Covidom from March 9, 2021, to November 1, 2021, included in the study.

A total of 81,634 patients were registered in the Covidom solution from March 9, 2020, to November 1, 2021, of which 60,073 (73.6%) met our inclusion criteria (Figure 1). Of the 60,073 patients, 51,813 (86.3%) were included as outpatients and 8260 (13.7%) were included at hospital discharge. Most outpatients were registered by GPs (28,064/51,813, 54.2%), followed by registrations after a hospital consultation (15,148/51,813, 29.2%), and finally after calling EMS (8601/51,813, 16.6%; not shown in Figure 1). The inclusion questionnaire was completed by 66.4% (34,426/51,813) of outpatients and by 63.8% (5272/8260) of patients included at hospital discharge. The median duration of the daily monitoring questionnaires was 27 (IQR 15-29) days after symptom onset. Patients included at hospital discharge were less likely to answer daily monitoring questionnaires during the 30-day follow-up period (6281/8260, 76.0%) than outpatients (44,860/51,813, 86.6%). Follow-up questionnaires were completed by 37.3% (22,425/60,073) of the patients on day 14 and by 11.8% (7073/60,073) on day 30.

Patients included at hospital discharge were more frequently male and older than those included during initial outpatient management, with 47.9% (2469/5155) versus 37.7% (12,797/33,940; *P*<.001) of male patients and an average age of 48.7 (SD 16.6) years versus 44.2 (SD 14.2 years; *P*<.001; Table 1), respectively. Among patients who completed the inclusion questionnaire, patients at hospital discharge also had significantly more comorbidities when compared with outpatients (Table 2), with 55.7% (2871/5155) versus 48.1% (16,307/33,940; *P*<.001) being overweight or obese, 20.0% (1052/5272) versus 12.3% (4247/33,940; *P*<.001) having hypertension, 10.9% (577/5272) versus 5.0% (1725/34,426; *P*<.001) with diabetes, 3.9% (207/5272) versus 2.0% (673/34,426; *P*<.001) with heart failure, 3.6% (191/5272) versus 1.2% (402/34,426; *P*<.001) with chronic renal disease, 3.3% (173/5272) versus 1.2% (399/34,424; *P*<.001) with cancer, and 3.0% (159/5272) versus 1.8% (615/34,426; *P*<.001) with chronic obstructive pulmonary disease, respectively. The rate of asthma did not significantly differ between patients at hospital discharge and outpatients (676/5272, 12.8% vs 4346/34,426, 12.6%; *P*=.79).

We found a corresponding match in the AP-HP data warehouse either for RT-PCR or hospitalization for 18% (10,815/60,093) of patients.

**Figure 2:**
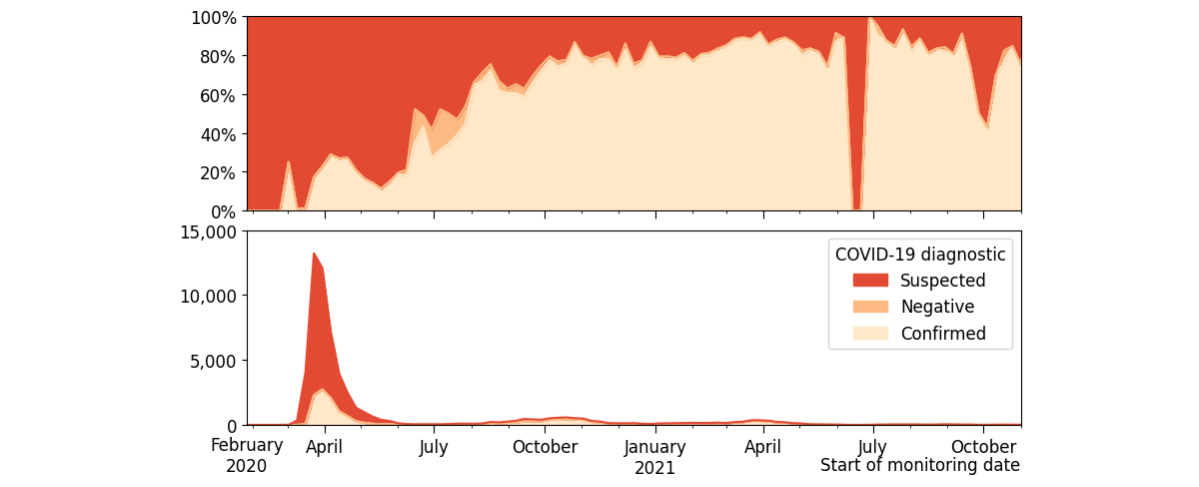
Proportion (top) and number (bottom) of Covidom inclusions over time, per the confirmed COVID-19 diagnostic.

Figure 2 shows the number and proportion of patients included in Covidom based on their COVID-19 confirmation test results. The patients were initially almost exclusively suspected COVID-19 cases until April 2020. After this date, the proportion of confirmed cases steadily increased, reaching over 80% (6697/8312) from October 2020. Negative patients were rare, representing <5% of the inclusions most of the time. Most patients were included in the first semester of 2020 (48,361/60,073, 80.5%), followed by 12.1% (7279/60,073) in the second semester and 6.6% (3969/60,073) and 0.8% (464/60,073) in the first and second semesters of 2021, respectively.

**Table 1:** General characteristics of all included patients and comorbidities and risk factors of patients who answered the inclusion questionnaire.

General characteristics of all included patients			Outpatients (n=51,813)		At hospital discharge (n=8260)		Total (n=60,073)		*P* value
**Sex, n (%)**									<.001
	Female (n=39,648)	21,584 (62.8)		2798 (53.1)		24,382 (61.5)			
	Male (n=39,648)	12,797 (37.2)		2469 (46.9)		15,266 (38.5)			
**Age (years), mean (SD)**			44.2 (14.2)		48.7 (16.6)		44.8 (14.6)		<.001
	18-45, n (%)	19,177 (55.7)		2342 (44.4)		21,519 (54.2)			
	45-65, n (%)	12,592 (36.6)		2037 (38.6)		14,629 (36.9)			
	>65, n (%)	2657 (7.7)		893 (16.9)		3550 (8.9)			

**Table 2:** Risk factor of patients who answered the inclusion questionnaire.

Patients who answered the inclusion questionnaire				Outpatients (n=34,426)		At hospital discharge (n=5272)		Total (n=39,698)		*P* value	
**Risk factors**											
	BMI (n=39,095), median (IQR)			24.8 (22.0-28.4)		25.8 (22.8-29.4)		24.9 (22.1-28.7)		<.001	
	**Weight, n (%)**										<.001
		Normal weight (BMI ≤25 kg/m^2^; n=39,095)	17,633 (52.0)		2284 (44.3)		19,917 (50.9)				
		Overweight (BMI 25-30 kg/m^2^; n=39,095)	10,075 (29.7)		1685 (32.7)		11,760 (30.1)				
		Obesity (BMI >30 kg/m^2^; n=39,095)	6232 (18.4)		1186 (23.0)		7418 (19.0)				
	Patient labeled “high risk” at inclusion, n (%)			13,307 (38.7)		2971 (56.4)		16,278 (41.0)		<.001	
	Current tobacco use, n (%)			6038 (17.5)		652 (12.4)		6690 (16.9)		<.001	
	Number of years smoking (n=6675), median (IQR)			15.0 (9.0-25.0)		15.0 (8.0-25.0)		15.0 (9.0-25.0)		.90	
	Cigarettes per day (n=6692), median (IQR)			10.0 (5.0-15.0)		10.0 (5.0-15.0)		10.0 (5.0-15.0)		.14	
**Main comorbidities, n (%)**											
	Asthma			4364 (12.7)		676 (12.8)		5040 (12.7)		.79	
	Hypertension			4247 (12.3)		1052 (20.0)		5299 (13.3)		<.001	
	Diabetes			1725 (5.0)		577 (10.9)		2302 (5.8)		<.001	
	Heart failure			673 (2.0)		207 (3.9)		880 (2.2)		<.001	
	Chronic obstructive pulmonary disease			615 (1.8)		159 (3.0)		774 (1.9)		<.001	
	Coronary artery disease			481 (1.4)		117 (2.2)		598 (1.5)		<.001	
	Cancer under treatment (n=39,696)			399 (1.2)		173 (3.3)		572 (1.4)		<.001	
	Chronic renal disease			402 (1.2)		191 (3.6)		593 (1.5)		<.001	
**General symptoms, n (%)**											
	Fatigue			29,663 (86.2)		4516 (85.7)		34,179 (86.1)		.34	
	Temperature ≥38.5 °C			16,816 (48.8)		2936 (55.7)		19,752 (49.8)		<.001	
	Shivers			18,624 (54.1)		2724 (51.7)		21,348 (53.8)		.001	
	Myalgia			18,771 (54.5)		2602 (49.4)		21,373 (53.8)		<.001	
**Respiratory symptoms, n (%)**											
	Cough			21,455 (62.3)		3242 (61.5)		24,697 (62.2)		.26	
	Shortness of breath			16,389 (47.6)		2915 (55.3)		19,304 (48.6)		<.001	
	Chest pain			8580 (24.9)		1298 (24.6)		9878 (24.9)		.65	
	Chest oppression			8833 (25.7)		1295 (24.6)		10,128 (25.5)		.09	
**Gastrointestinal symptoms, n (%)**											
	Anorexia			13,374 (38.8)		2469 (46.8)		15,843 (39.9)		<.001	
	Nausea, vomiting, or both			8253 (24.0)		1450 (27.5)		9703 (24.4)		<.001	
	Diarrhea			12,040 (35.0)		1906 (36.2)		13,946 (35.1)		.10	
**Neurological symptoms, n (%)**											
	Anosmia			11,299 (32.8)		1623 (30.8)		12,922 (32.6)		.003	
	Ageusia			11,119 (32.3)		1720 (32.6)		12,839 (32.3)		.64	
**Cutaneous symptoms, n (%)**											
	Rash			3159 (9.2)		394 (7.5)		3553 (9.0)		<.001	
	Chilblains			631 (1.8)		92 (1.7)		723 (1.8)		.70	
	Conjunctivitis			2439 (7.1)		289 (5.5)		2728 (6.9)		<.001	

### Primary Outcome

A total of 285,496 alerts were handled by the regional control center for the included patients over the study period (Table 3), with a median of 4 (IQR 2-8) alerts per patient. Of these 285,496 alerts, 258,200 (90.4%) had normal priority and 27,296 (9.6%) had high priority. Overall, 24.7% (14,858/60,073) of patients generated at least 1 high-priority alert, 74.5% (44,762/60,073) generated at least 1 normal-priority alert, and 10.6% (6381/60,073) generated no alert. The remaining 14.9% (8930/60,073) of patients never completed a monitoring questionnaire and, thus, could not trigger alerts.

**Figure 3:**
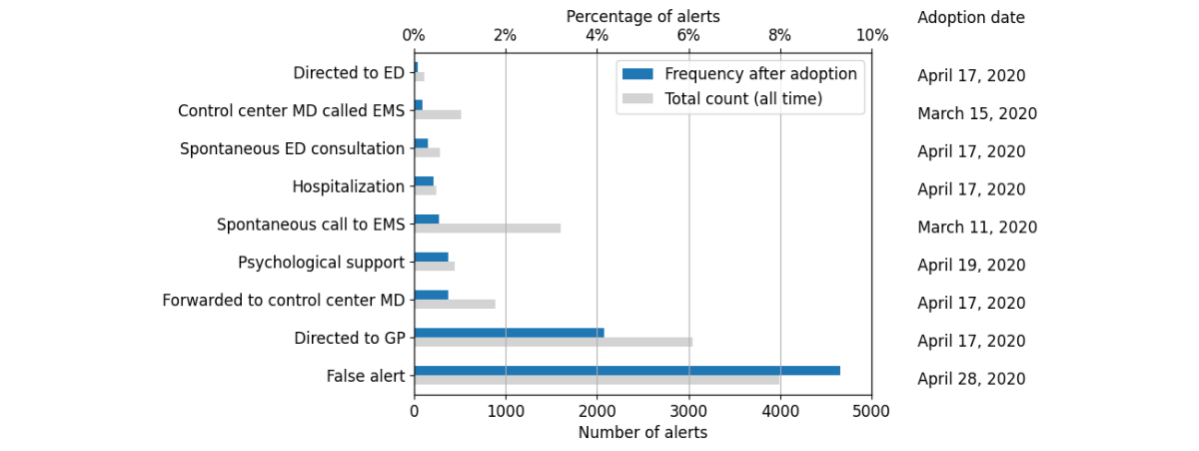
Alert category frequency after adoption, first use date, and total occurrence counts. Note: category frequencies are computed starting 30 days after the first occurrence of that alert (right), except for “Control center MD called EMS,” which corresponds to the whole study period. ED: emergency department; EMS: emergency medical services; GP: general practitioner; MD: medical doctor.

Outpatients were more likely to trigger alerts, with 75.7% (39,223/51,813) of patients having at least 1 alert compared with 67.1% (5539/8260) of patients included at hospital discharge. However, the rate of high-priority alerts did not seem to differ between patients included at hospital discharge (2104/8260, 25.5%) and outpatients (12,754/51,813, 24.6%). The main alert classifications reported by RMRs are shown in Figure 3. The most common alert category was false alerts, accounting for 9.3% (3992 alerts by 1852 patients) of all alerts during the study period. Of the 9169 patients included after the introduction of false alert labels, 7261 (79.2%) never generated false alerts, most patients triggering a false alert only had a single occurrence (n=1431, 15.6%), and only 1.6% (n=147) of patients had ≥3 false alerts. The most frequent alert escalation was patients being addressed to their GP in 4.2% of alerts (3043 alerts generated by 1030 patients), followed by 0.7% of alerts forwarded to the control center physician (888 alerts by 213 patients), 0.1% of alerts for which the patient was directed to the ED (112 alerts by 26 patients), and 0.2% of alerts for which the control center physician called the EMS (518 alerts by 484 patients).

**Table 3:** Number and duration of alerts (n=285,496).

Priority	Outpatients, n (%)	At hospital discharge, n (%)	Total, n (%)	Response time, median (IQR)	Handling time, median (IQR)
All	250,317 (87.7)	35,179 (12.3)	285,496 (100)	8 minutes 30 seconds (1 minute 24 seconds to 1 hour 11 minutes 13 seconds)	38 minutes 45 seconds (10 minutes 54 seconds to 3 hours 23 minutes 30 seconds)
Normal	227,210 (79.6)	30,990 (10.9)	258,200 (90.4)	10 minutes 34 seconds (1 minute 32 seconds to 1 hour 26 minutes 11 seconds)	45 minutes 55 seconds (11 minutes 13 seconds to 3 hours 52 minutes 14 seconds)
High	23,107 (8.1)	4189 (1.5)	27,296 (9.6)	2 minutes 57 seconds (0 minutes 55 seconds to 10 minutes 0 seconds)	17 minutes 32 seconds (09 minutes 6 seconds to 47 minutes 49 seconds)

### Safety of Covidom

High-priority alerts had a median time to first response of 3 (IQR 1-10) minutes and were handled within a median time of 18 (IQR 9-48) minutes. Normal-priority alerts had a median time to first response of 11 minutes (IQR 2 minutes to 1 hour 26 minutes) and a handling time of 46 minutes (IQR 11 minutes to 3 hours 52 minutes; Table 3). The highest level of escalation from the control center, which required calling EMS, was reached significantly less frequently for normal-priority alerts (283/258,200, 0.1%) than for high-priority alerts (235/27,296, 0.9%; *P*<.001).

Of the 60,073 patients, 2527 (4.2%) had clinical worsening within 1 month after symptom onset or hospital discharge. Among 51,813 outpatients, 1796 (3.5%) were hospitalized and 118 (0.2%) died, together accounting for a clinical worsening rate of 3.6% (n=1866; 95% CI 3.4%-3.8%). For 8260 patients included at hospital discharge, 633 (7.7%) were rehospitalized and 47 (0.6%) died, which together represents a clinical worsening rate of 8.0% (n=661; 95% CI 7.4%-8.6%).

Among 2527 patients with clinical worsening, 947 (37.5%) had a known date of hospital admission or death. Among these 947 patients, 180 (19.0%) triggered a high-priority alert within the 5 days before the worsening, 258 (27.2%) triggered a normal-priority alert, 35 (3.7%) triggered no alert, and the remaining 476 (50.3%) did not participate in monitoring in these 5 days. Of the 35 patients who triggered no alert in the 5 days preceding the clinical worsening, 35 were hospitalized, including 1 who died. These patients had an average age of 51.1 (SD 15.6) years, were mostly female (25/35, 71%), and answered a median of 3 (IQR 2-5) daily questionnaires out of a maximum of 6 (1 on the day of worsening and 1 each on the 5 preceding days).

Among the 1580 patients who experienced clinical worsening with no known worsening date, 43.2% (n=683) triggered at least 1 high-priority alert, 41.3% (n=653) triggered at least 1 normal-priority alert, 3.9% (n=61) never triggered an alert, and 11.5% (n=181) did not participate in any monitoring.

During alert responses, patients reported having been hospitalized in 0.4% of alerts (120 patients), having spontaneously called EMS in 0.5% of alerts (696 patients), and having spontaneously consulted the ED in 0.3% of alerts (94 patients). Finally, the patients requested psychological support in 0.7% of alerts (177 patients).

The 13,204 patients who responded to either of the follow-up questionnaires at 14 days and 30 days (13,204/60,073, 22.0% of the cohort) reported a total of 27,961 medical contacts, of which 12,037 (43.0%) were remote appointments with a GP, 10,688 (38.2%) were consultations at a GP office, 2573 (9.2%) were calls to EMS, 2145 (7.7%) were ED visits, and 518 (1.9%) were home visits by GPs. In total, 65.8% (8690/13,204) of respondents reported having consulted outside the Covidom solution during their Covidom monitoring period.

### Cost of the Covidom Solution

The total cost of the Covidom solution corresponded to an average of €54.33 (US $1= €0.8614) per patient, with 48.4% (€26.31/€54.33) being staff costs, 38.5% (€20.94/€54.33) information technology costs, and 13% (€7.09/€54.33) structural and administration costs.

The average out-of-hospital costs were €24.55 per patient (Table 4), with 518 EMS dispatches being the most costly item (€490,546/€1,475,054, 33.3%). To compute hospital costs of the worsened patients, 54.9% (1387/2527) of patients were matched with the AP-HP data warehouse, 1006 (39.8%) with at least 1 hospital stay in the 30 days after starting the follow-up, and 962 (38.0%) with an available diagnosis classification (Table 5). These hospitalized patients had a median hospital stay of 3 (IQR 1-9) days, and 1.1% (11/962) of them stayed in an intensive care unit for 3 (IQR 1.5-3.5) days. The cost of these hospitalizations was €7001 on average per patient.

**Table 4:** Out-of-hospital health care use by Covidom patients, as reported in follow-up questionnaires or initiated by remote monitoring responders, and associated costs.

Type of out-of-hospital care	Patients, n	Per patient, median (IQR)	Total direct costs (€^a^)
Number of teleconsultations	12,037	2 (1-2)	300,950
In-person general practitioner consultation	10,688	1 (1-2)	320,640
Call to emergency medical services regulation center	2573	1 (1-2)	41,168
Emergency department visit	2145	1 (1-1)	321,750
Emergency medical services dispatch	518	0 (0-0)	490,546

^a^US $1= €0.8614; total costs per patient: €24.55.

**Table 5:** Cost of hospitalization of Covidom patients (n=962).

Covidom patients with diagnosis-related group classification in Assistance Publique-Hôpitaux de Paris data warehouse	Value
Patients in intensive care unit, n (%)	11 (1.1)
In-hospital deaths, n (%)	62 (6.4)
Hospitalization duration (days), median (IQR)	3 (1-9)
Intensive care duration (days), median (IQR)	3 (1.5-3.5)
Hospitalization cost (€^a^), median (IQR)	4249 (1180-12,427)
Total hospitalization costs (€)	6,735,146
Total costs per hospitalized patient (€)	7001

^a^US $1= €0.8614.

### Comparison Among Mild COVID-19 Cases Presenting at AP-HP EDs

**Figure 4:**
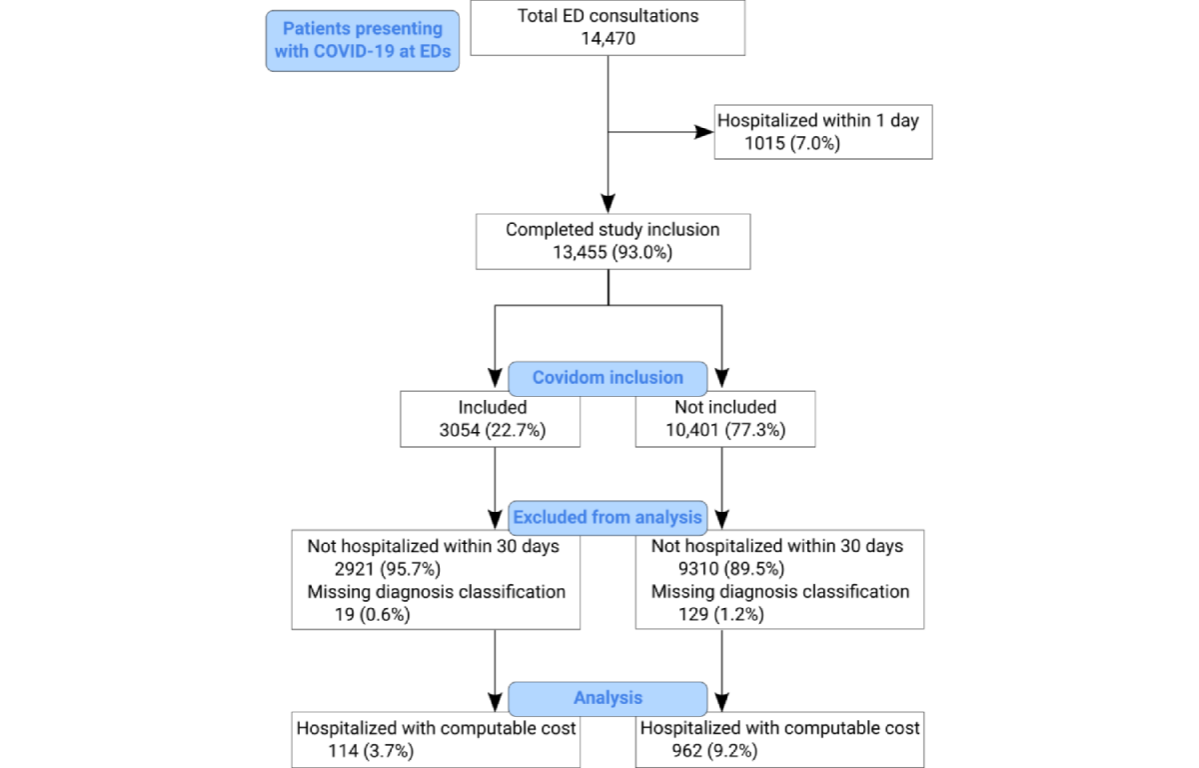
Flowchart of patients consulting at Assistance Publique-Hôpitaux de Paris emergency departments for mild COVID-19. ED: emergency department.

Of the 13,455 patients initially consulting at AP-HP EDs for mild COVID-19 (Figure 4), 3054 (22.7%) were included in Covidom. Included patients were more frequently female (1868/3054, 61.2%) than patients who were not registered (5141/10,401, 49.4%) and had a similar age with a mean of 43.6 (SD 14.1) years versus 44.0 (SD 9.8) years. Among all patients with mild COVID-19 presenting at AP-HP EDs, 9.1% (1224/13,455) were later hospitalized. After the removal of 148 patients without diagnosis classification information, for whom the cost of hospitalization was therefore unknown, 1076 patients were left for analysis. Of these 1076 patients, 114 (10.6%) were included in the Covidom solution. Age and sex were distributed similarly regardless of Covidom inclusion, with 38.6% (44/114) of female patients among Covidom patients against 39.0% (375/962) of female patients among non-Covidom patients, and mean ages were 56.6 (SD 13.6) years and 57.5 (SD 19.7) years, respectively. Patients registered in Covidom were hospitalized later, with a median time between consultation and hospitalization of 5 (IQR 3-7) days versus 4 (IQR 3-7) days. Hospitalization costs were lower for patients included in Covidom, with a median cost of €3202 (IQR €1575-€5512) versus €4134 (IQR €2115-€7148; Table 6).

**Table 6:** Comparison of characteristics and hospitalization cost of patients with mild COVID-19 initially consulting at Assistance Publique-Hôpitaux de Paris emergency departments between those included and those not included in Covidom. (n=1076)

		Included in Covidom (n=114)	Not included in Covidom (n=962)	Total (n=1076)	*P* value	
**Sex, n (%)**						.98
	Female	44 (38.6)	375 (39.0)	419 (38.9)		
	Male	70 (61.4)	587 (61.0)	657 (61.1)		
Age (years), mean (SD)		56.6 (13.6)	57.5 (19.7)	57.4 (19.1)	.54	
Days to hospitalization, median (IQR)		5 (3-7)	4 (3-7)	4 (3-7)	.03	
Hospitalization cost (€^a^), median (IQR)		3202.0 (1575.0-5511.8)	4146.0 (2132.2-7141.0)	3790.0 (2100.0-7106.0)	.01	

^a^US $1= €0.8614.

### Satisfaction of Patients

**Figure 5:**
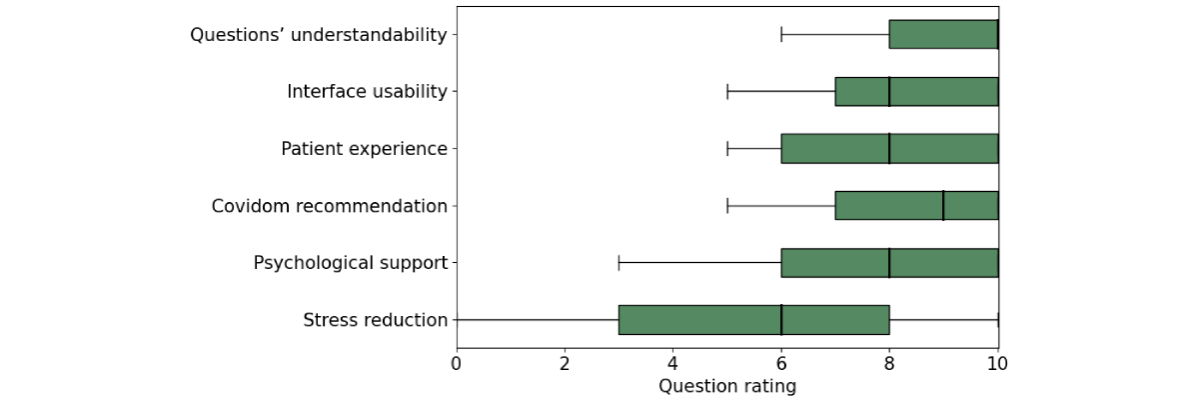
Median and quartiles of satisfaction grading for Covidom questions. Vertical bars display median scores, boxes represent IQR, and whiskers represent minimum-maximum scores.

In total, 17% (10,219/60,073) of patients answered the satisfaction questions as part of the follow-up questionnaires, and the results are presented in Figure 5. Respondents rated highly the understandability of the monitoring questionnaire, with a median score of 10 (IQR 8-10) out of 10, and the interface usability, with a median score of 8 (IQR 7-10). Patients rated highly their overall experience with Covidom, with a median score of 8 (IQR 6-10), and their likelihood of recommending Covidom, with a median score of 9 (IQR 7-10). Finally, patients also felt supported by the Covidom solution, rating psychological support at a median of 8 (IQR 6-10), although patients often remained stressed by the COVID-19 situation, rating the stress reduction thanks to Covidom at a median of 6 (IQR 3-8).

## Discussion

### Principal Findings

This study provides an evaluation of the Covidom telemonitoring solution after 18 months of activity, looking back on over 60,000 patients that were monitored and their outcomes and 285,000 alerts handled as well as quantifying the effectiveness, safety, and cost of this solution. Covidom was mostly used to monitor symptoms in outpatients with mild COVID-19 and less frequently after COVID-19–related hospitalization. Our results suggest that Covidom was effective in its task of monitoring and reassuring patients with mild COVID-19, was overall safe with a great responsiveness in case of high-priority alerts and with very few patients presenting with clinical worsening without having previously triggered an alert. Nevertheless, the number of consultations outside Covidom were substantial, and although our results suggest a lower cost per hospitalized patient, the overall cost of the solution per patient remains high.

Patients had high adherence to the monitoring system, with over two-thirds of patients having completed the medical questionnaire at inclusion and over half of the patients having completed monitoring questionnaires for the full duration of 30 days after first symptoms onset. Follow-up questionnaires at days 14 and 30 of monitoring had a lower response rate, which may be attributed to patients disengaging with the solution after feeling better, as Covidom’s emphasis was set on monitoring rather than research.

With >250,000 alerts handled, when accounting for false alerts, and an estimated 2114 medical consultations over the phone, the solution may have reduced the workload of hospitals, GPs, and EMS call centers by providing medical and practical information and reassuring patients. Covidom referred patients to in-person consultations only when it appeared to be necessary, thereby relieving the health care facilities in the region as much as possible. This effect may seem corroborated by a later hospital presentation when compared with patients with mild COVID-19 outside Covidom. By monitoring patients at home, the telemonitoring solution helped preserve the physical isolation of infectious patients during the pandemic period. Covidom also provided reassurance to many patients who were stressed, especially in the first months of the pandemic. However, close to 66% (8690/13,204) of patients who answered the follow-up questionnaires still reported consulting outside of Covidom. Another surprising result of this study was the higher rate of patients being directed to their GP rather than being managed by the control center physician. Although this practice seems opposite to the solution’s goal of alleviating the health care system, it is worth noting that the solution was implemented for a much longer period than the lockdowns in the area and that phone consultations remain limited for a number of diagnoses.

Most cases of clinical worsening showed early warning signs through the alert system and included patients who were at least moderately compliant with the Covidom monitoring questionnaires. Furthermore, the rates of patient worsening and EMS dispatches were higher for high-priority alerts than for normal-priority alerts, which suggests that the targeting of high-priority alerts is relevant. The priority with which these alerts were handled allows for much faster first response times, with >75% (20,472/27,296) of the high-priority alerts receiving a first response in ≤10 minutes. However, a significant fraction of hospitalizations and deaths of the registered patients occurred among patients who did not actively participate in the monitoring after confirming their registration.

The low number of hospitalizations and deaths missed by the system was achieved through a high alert sensitivity, which resulted in a low precision with numerous alerts (close to 1 in 11) not corresponding to patient clinical worsening. There was a low proportion of people causing repeated false alerts, which suggests a learning curve in measuring vitals and answering monitoring questionnaires appropriately. This was also a deliberate choice to maximize the safety of the solution, and any adjustments of the alert algorithm always sought to maintain the risk of missing a patient’s clinical worsening to a minimum.

Overall, the cost of the Covidom solution was high, with an average of €54 per patient for >60,000 patients. The hospitalization cost of Covidom patients seems lower than that of patients not registered in Covidom, although this difference may not be sufficient for the solution to be cost neutral. Nevertheless, cost offsets were not the primary goal in the context of an emerging infectious disease pandemic at a time (mid-2020) when little was known about the disease. The high number of consultations outside Covidom suggests that a telemonitoring solution at the implemented scale has limitations for long-term use. Outside the context of care saturation, and once risk factors and illness evolution are well understood, a more targeted public could be selected for registration in such a monitoring program. Indeed, disclosing medical information and answering daily monitoring questionnaires already seemed demanding to some patients and medical institutions in the context of a pandemic, and the acceptability of the solution probably decreased further over time, along with the risk and uncertainty surrounding the disease. This seems to be corroborated by the inclusions in Covidom decreasing strongly over time, with only 19.5% (11,712/60,073) of inclusions after June 2020, although this may also be attributed to patients having access to tests and information without having to go through a medical practitioner and, thus, without the opportunity to be registered in Covidom. The Covidom solution stopped its activity on March 31, 2022.

This type of telemonitoring has already been implemented to monitor patients discharged from hospital [17], but it was novel for infectious outpatients in the context of an acute epidemic. Similar telemedicine solutions have been developed simultaneously at a much smaller scale (313 patients) in Spain [18] or to monitor patients isolating in hotels rather than at home in Italy and Canada [19,20]. An analysis of similar remote monitoring in a community setting in the United States with a much simpler 2-question system [21] seems to indicate that monitoring may be associated with lower mortality and more or earlier presentation to the hospital in the context of a strained health care system. Without accounting for as many factors, our data seem to indicate that monitoring is associated with a lower hospitalization cost and a later presentation at the hospital. As underlined in previous work [22], such solutions could become an integral part of health care options, likely targeted to more specific populations, such as patients at risk of isolation or presenting risk factors. The safety and value of the Covidom telemonitoring solution needs to be put in perspective with a number of factors. First, requiring an initial medical consultation for registration limited the reduction in recourse to care and may also have limited the solution to populations that are more likely to consult a physician. Patients also needed to be at ease with recent technologies and possess a smartphone, tablet, or computer, which potentially excluded certain more populations considered more vulnerable, such as older or economically disadvantaged patients. A small number of minors were included in Covidom; however, this evaluation excluded this peculiar population. Another concern with respect to safety is the high number of hospitalized patients who did not adhere to the monitoring, with 50% (476/947) of hospitalized patients with a known hospitalization date not having completed any monitoring questionnaires in the 5 days preceding their hospital admission. This makes it impossible for the Covidom solution to detect those worsening cases early on; however, it is difficult to identify the cause of this phenomenon, which could range from having another type of monitoring to more vulnerable patients being less at ease with technology or even patients being too ill to participate in monitoring.

### Limitations

This study could not provide a high level of evidence because of its observational nature and the lack of a control group. Owing to the urgency of deploying the solution in early March 2020, no formal evaluation mechanism was put in place. Other data that would be useful for computing the efficiency of the alert system, such as hospitalization dates of patients, were only available for 37% (947/2527) of our hospitalized patients. In addition, the peculiar context of a sudden pandemic and lack of a comparable cohort of patients with mild COVID-19 without monitoring means that there is no clear point of comparison for a cost-effectiveness analysis. Although future work on health care reimbursement data should provide a more definitive answer, the hospitalization cost comparison presented in this study provides some insight.

Diagnostic confirmation of the included patients remained low in the first months because of the limited access to RT-PCR tests in France until June 2020 and the prioritization of severe COVID-19 cases over mild cases in accessing tests. During the summer months of 2020, from the end of the first lockdown in May to the uptake of the epidemic in September, the number of inclusions remained low (n=2049 from May 11, 2020, to August 31, 2020), which explains the relatively high number of negative cases included. Tests may also yield false positives, particularly during the early months of the pandemic.

As 78% (46,869/60,073) of patients did not answer follow-up questionnaires, there was a risk of missing a significant portion of the health care cost data and a risk of self-selection bias among respondents to the satisfaction questions. This bias could cause patients that were more anxious, more satisfied, or with worse forms of the disease to be more likely to answer. Another potential bias is that data on RT-PCR testing and hospitalizations may be incomplete, as the AP-HP data warehouse covers only around half of the hospitals in the area, which is also reflected by the frequently unavailable hospitalization dates. It will be possible to complete these data in future work by additionally matching health care reimbursement data.

### Conclusions

The main goal of this study was to provide an overall evaluation of the Covidom telemonitoring solution 18 months after its implementation. Our results seem to indicate that Covidom’s efforts to alleviate hospitals and practitioners were quickly implemented and reasonably safe, at a time when saturation of care providers was a major concern of all health care systems, although the need for this solution on a longer time scale is debatable. Covidom was made possible by the collaboration of many hospitals, EMS call centers, and GPs in the region, all registering patients with suspected or confirmed COVID-19 in the monitoring program. This study suggests that providing monitoring, medical support, and reassurance to patients, even for a poorly known disease at the time, is possible.

## Appendix

Multimedia Appendix 1 Daily monitoring questions and answers, translations and original French, and determination of alert priority from answers.

Multimedia Appendix 2 Calculation of out-of-hospital costs.

Multimedia Appendix 3 User satisfaction questions, translations and original French.

Multimedia Appendix 4 Contributors to Covidom, including data science, scientific, and steering committees; remote monitoring responders; supervising physicians; and including physicians.

## Abbreviations

AP-HP: Assistance Publique-Hôpitaux de Paris
ED: emergency department
EMS: emergency medical services
GP: general practitioner
RMR: remote monitoring responder
RT-PCR: reverse transcription polymerase chain reaction

## Data Availability

The data sets generated and analyzed during this study are not publicly available because of privacy and confidentiality protection but are available upon reasonable request. Deidentified data sets of Covidom inclusions and alerts over time are available under an open license on the French Government open data platform [23].
