# Supplementary material for: Effectiveness and Medicoeconomic Evaluation of Home Monitoring of Patients With Mild COVID-19: Covidom Cohort Study
Source: J Med Internet Res. 2023 Jun 23;25:e43980. doi: 10.2196/43980 (PMC10337320; doi:10.2196/43980)
Supplement: Multimedia Appendix 1 [file jmir_v25i1e43980_app1.pdf]

Appendix 1: Daily monitoring questions and answers, translations and original French, and determination of alert priority from answers

High-priority alerts were triggered for any bold red answer, or for two or more orange underlined answers. Questionnaires with a single orange underlined answer generated a normal priority alert, while questionnaires with only unmarked answers generated no alert. We also list below the table the changes in alert handling with the dates at which they occurred, where the table presents the most up-to-date versions of questions and rules.

Question	Question phrasing – answer template	Possible answer values				
**Temperature**	Take your temperature (in °C)<br>- My temperature is … °C	lower than 35.5	between 35.5 and 39.1	between 39.1 and 40.0	**higher than 40**	
	Prenez votre température (en °C)<br>- Ma température est … °C	inférieure à 35,5	comprise entre 35,5 et 39,0	comprise entre 39,1 et 40,0	**supérieure à 40**	
**Dyspnea**	Tick the box that best fits your respiratory discomfort (dyspnea)	None	Slight	Moderate	**Severe**	**Very Severe**
	Cochez la case décrivant le mieux votre gêne respiratoire (dyspnée)	Absente	Faible	Modérée	**Forte**	**Majeure**
**Heart rate**	Measure your heart rate (in beats per minute or bpm)<br>- My heart rate is … bpm	less than 100	between 100 and 120	**higher than 120**		
	Mesurez votre fréquence cardiaque (en battements par minute ou bpm)<br>- Ma fréquence cardiaque est … bpm	inférieure à 100	comprise entre 100 et 120	**supérieure à 120**		
**Respiratory rate**	Measure your respiratory rate (in cycles per minute)<br>- My respiratory rate is … cycles/min	less than 20	between 20 and 30	**higher than 30**		
	Mesurez votre fréquence respiratoire (en cycles par minutes)<br>- Ma fréquence respiratoire est … cycles/min	inférieure à 20	comprise entre 20 et 30	**supérieure à 30**		
**Oximeter**	Do you own a pulse oximeter?	Yes	No			
	Disposez-vous d'un saturomètre ?	Oui	Non			
**Saturation** *	Measure your saturation (expressed as a percentage)<br>- Ma saturation is between …	**85 and 90%**	91 and 94%	95 and 100%		
	Mesurez votre saturation (exprimée en pourcentage)<br>- Ma saturation est comprise entre …	**85 et 90%**	91 et 94%	95 et 100%		

Question	Question phrasing – answer template	Possible answer values										
**Shivers**	Have you had shivers since the last questionnaire or the last medical contact?	Yes	No									
	Avez-vous eu des frissons depuis que vous avez rempli le dernier questionnaire ou depuis le dernier contact médical ?	Oui	Non									
**Faintness**	Have you fainted since the last questionnaire or the last medical contact?	Yes	No									
	Avez-vous fait un malaise depuis que vous avez rempli le dernier questionnaire ou depuis le dernier contact médical ?	Oui	Non									
**Isolation**	Do you manage to stay confined in good material conditions (ability to isolate from other family members, obtaining groceries, and eating)?	Yes	No									
	Arrivez-vous à poursuivre votre confinement dans de bonnes conditions matérielles (possibilité de vous isoler des autres membres de la famille, d'avoir des courses alimentaires et de vous nourrir) ?	Oui	Non									
**Nausea**	Do you experience nausea or vomiting that prevents you from eating?	Yes	No									
	Avez-vous des nausées ou vomissement qui vous empêchent de vous alimenter ?	Oui	Non									
**Distress**	Can you rate your state of psychological distress on a scale from 0 to 10?	0	1	2	3	4	5	6	7	8	9	10
	Pouvez-vous noter de 0 à 10 votre détresse psychologique ?											
**Call back ****	Do you want to be called back for psychological support?	**Yes**	No									
	Souhaitez-vous être appelé pour un soutien psychologique ?	**Oui**	Non									
**Degradation**	Do you feel worse than yesterday?	Yes	No									
	Allez-vous moins bien qu'hier ?	Oui	Non									

* The saturation question was only asked if the pulse oximeter answer is yes

** The callback question was only asked if the psychological distress is rated 3/10 or higher

Changes in alert handling were:

- March 31st 2020: Changed a temperature threshold from 38°C to 39°C to generate less false alerts, and changed dyspnea scale from 10 to 5 values to simplify questionnaire.
- April 22nd 2020: “Nausea”, “Call back”, and “Distress” questions added to questionnaire.
- May 12th 2020: Started only asking “Call back” if “Distress” answer is 3 or higher.
- May 16th 2020: “Degradation” question added to questionnaire.
- April 22nd 2020: “Temperature” answer under 35.5 stopped generating alerts, instead of high-priority alerts (except from May 1st to May 16th 2020, where the previous behavior was reverted), to limit false alerts from thermometer misuse.
