# Supplementary material for: Effectiveness and Medicoeconomic Evaluation of Home Monitoring of Patients With Mild COVID-19: Covidom Cohort Study
Source: J Med Internet Res. 2023 Jun 23;25:e43980. doi: 10.2196/43980 (PMC10337320; doi:10.2196/43980)
Supplement: Multimedia Appendix 2 [file jmir_v25i1e43980_app2.pdf]

Appendix 2: Out-of-hospital costs calculation

	**Unit cost (€[a])**	**Source**
Hospital Emergency Department (ED) visit for COVID-19, (min-max)	150 (80−250)	Parliamentary report [13]
Call to EMS regulation center (fixed)	16	Commissioned report [14]
EMS dispatch (fixed)	947	Commissioned report [14]
Outpatient consultation – medical doctor (min-max)	30 (20−50)	Social health insurance fee schedule
Telemedicine consultation (fixed)	25	French Ministry for Solidarities and Health Invoicing guide for telemedicine [15]

[a]US $1= €0.8614.
