# Supplementary material for: Effectiveness and Medicoeconomic Evaluation of Home Monitoring of Patients With Mild COVID-19: Covidom Cohort Study
Source: J Med Internet Res. 2023 Jun 23;25:e43980. doi: 10.2196/43980 (PMC10337320; doi:10.2196/43980)
Supplement: Multimedia Appendix 3 [file jmir_v25i1e43980_app3.pdf]

Appendix 3: User satisfaction questions, translations and original French

**Question's understandability**	Did you find the questions understandable?<br>Avez-vous trouvé les questions compréhensibles ?
**Interface usability**	How would you rate the Covidom interface?<br>Comment noteriez-vous l'interface Covidom ?
**Patient experience**	How would you rate this type of home care?<br>Comment noteriez-vous l'intérêt de ce mode de prise en charge à domicile ?
**Covidom recommendation**	Would you recommend this type of follow-up to your family members of friends?<br>Recommanderiez-vous un tel suivi à vos proches ou vos amis ?
**Psychological support**	Did you feel psychologically supported by Covidom?<br>Vous êtes-vous senti(e) soutenu moralement par Covidom ?
**Stress reduction**	Do you think Covidom helped reduce stress caused by the Covid-19 infection?<br>Pensez-vous que Covidom ait permis de diminuer votre niveau de stress lié à l'infection à Covid-19 ?
